# Neurobehavioral consequences of chronic intrauterine opioid exposure in infants and preschool children: a systematic review and meta-analysis

**DOI:** 10.1186/1471-244X-14-104

**Published:** 2014-04-08

**Authors:** Alex Baldacchino, Kathleen Arbuckle, Dennis J Petrie, Colin McCowan

**Affiliations:** 1 Division of Neuroscience, Medical Research Institute, University of Dundee, Ninewells Hospital and Medical School, Dundee DD1 9SY, UK; 2 Division of Population Health Science, Medical Research Institute, University of Dundee, Ninewells Hospital and Medical School, Dundee DD1 9SY, UK; 3 Centre for Health Policy, Melbourne School of Population and Global Health, University of Melbourne, Victoria, Australia; 4 Robertson Centre for Biostatistics, Institute of Health and Wellbeing, College of Medical, Veterinary and Life Sciences, University of Glasgow, Boyd Orr Building, Level 11, Glasgow G12 8QQ, UK

**Keywords:** Neuropsychology, Psychomotor, Cognition, Methadone, Opioids, Meta-analysis

## Abstract

**Background:**

It is assumed within the accumulated literature that children born of pregnant opioid dependent mothers have impaired neurobehavioral function as a consequence of chronic intrauterine opioid use.

**Methods:**

Quantitative and systematic review of the literature on the consequences of chronic maternal opioid use during pregnancy on neurobehavioral function of children was conducted using the Meta-analysis of Observational Studies in Epidemiology (MOOSE) and the Preferred Reporting Items for Systematic Reviews and Meta-Analysis (PRISMA) guidelines. We searched Cinahl, EMBASE, PsychINFO and MEDLINE between the periods of January 1995 to January 2012.

**Results:**

There were only 5 studies out of the 200 identified that quantitatively reported on neurobehavioral function of children after maternal opioid use during pregnancy. All 5 were case control studies with the number of exposed subjects within the studies ranging from 33–143 and 45–85 for the controls. This meta-analysis showed no significant impairments, at a non-conservative significance level of p < 0.05, for cognitive, psychomotor or observed behavioural outcomes for chronic intra-uterine exposed infants and pre-school children compared to non-exposed infants and children. However, all domains suggested a trend to poor outcomes in infants/children of opioid using mothers. The magnitude of all possible effects was small according to Cohen’s benchmark criteria.

**Conclusions:**

Chronic intra-uterine opioid exposed infants and pre-school children experienced no significant impairment in neurobehavioral outcomes when compared to non-exposed peers, although in all domains there was a trend to poorer outcomes. The findings of this review are limited by the small number of studies analysed, the heterogenous populations and small numbers within the individual studies. Longitudinal studies are needed to determine if any neuropsychological impairments appear after the age of 5 years and to help investigate further the role of environmental risk factors on the effect of ‘core’ phenotypes.

## Background

Substance abuse has been a global problem for many decades and in recent years there has been a significant increase in the numbers of people using opioids [1]. Opioid use was seen as a predominately male problem but today there are many women using opioids which could lead to an increase in problem pregnancies [2]. During pregnancy drugs will cross the placenta and can have an effect on the foetus. This effect is often hard to quantify as there are other aspects that could be considered as having a larger effect on child outcomes, for example, the quality of care or the environment [3]. Many studies examining the impact of opioid use during pregnancy on child outcomes have concentrated on treatment populations (methadone and/or buprenorphine) for recruitment as this group is easier to reach than heroin users [4]. Research has attempted to address birth problems, neonatal abstinence syndrome, mortality and co-morbidities as well as neuro-developmental issues in children sometimes with conflicting results [5]. There are many reports into neonatal abstinence syndrome and birth parameters but fewer reports on neuro-developmental issues surrounding prenatal exposure to opioids [6-10].

In the U.K. it is estimated that around 280,000 people use opioids and that around 30% are women [11,12]. In 2009/2010 925 pregnancies in Scotland reported drug misuse, a rate of 16.1 per 1,000 pregnancies, with opioids reported in 506 (55%) of these pregnancies [2]. Over half the pregnant mothers who report drug use are opioid dependent with consequential increase in risk to both mother and expected child.

Replacement prescribing with methadone and recently buprenorphine forms the main plank of medical treatment for opioid dependency in the United Kingdom, reflecting a comprehensive and evolving evidence base which consistently demonstrates the effectiveness of methadone in delivering positive outcomes in a complex and demanding population [13-15]. Properly prescribed and adequately supported, methadone prescribing achieves harm reduction outcomes in opioid dependent patients [16,17]. It is also associated with reduced mortality and improved quality of life [18]. The duration and dosage of methadone was also closely observed to be relevant factors in treatment outcomes with longer duration and higher dosages showing positive outcomes [19-21].

In contrast, some neuropsychological studies of chronic methadone users have identified deficits in executive function measures. These have included impairments in cognitive flexibility [22,23], in strategic planning [24,25] and decision making [26]. Other studies found no clear deficits when comparing the performance of healthy controls, with that of opioid abstinent or methadone users [27,28]. The accumulated literature tends to assume that neuropsychological function is commonly impaired as a consequence of chronic methadone use justifying an abstinence agenda with premature termination of methadone treatment [29]. Furthermore a recent meta-analysis on the neuropsychology of chronic opioid use suggested impairment in verbal working memory, cognitive impulsivity (risk taking) and cognitive flexibility (verbal fluency) with a medium effect size [30].

An early study has suggested that methadone-exposed children have better birth outcomes compared to heroin-exposed children, suggesting that opioid treatment during pregnancy is beneficial for the neonate [31]. Despite evidence of the beneficial effects of methadone in the care of pregnant opioid-dependent women, approximately half of all infants prenatally exposed to methadone require medical treatment for neonatal abstinence syndrome [32]. Accordingly, there are risks associated with prenatal exposure to methadone or buprenorphine [33]. A recent Cochrane systematic review identified four trials comparing methadone in pregnancy with buprenorphine in three studies and oral slow-release morphine in the other [34]. Patients using methadone had lower dropout rates than for the other treatment options but there were no differences in neo-natal abstinence syndrome in the trials. Infant birth weight was higher for buprenorphine users in two trials but no different in the other two trials. Women on slow-release morphine were less likely to also use heroin in the third trimester than methadone users. The authors highlighted the lack of available evidence to inform treatment decisions for pregnant women with opioid dependence.

The literature pertaining to the long-term developmental effects of prenatal methadone and buprenorphine exposure is relatively sparse and contradictory [5]. While some studies report no long-term effects [35-37] others report reduced performance on tests of cognitive development [38-41].

While this literature, together with the neurobehavioral effects of intrauterine opioid use on children, has been reviewed by Konijnenberg and Melinder (2011) [5], Whitham (2012) [42] and Hutchings (1987) [43], traditional narrative reviews typically assume statistically significant group differences to be evidence for cognitive and/or psychomotor impairment, without giving due consideration to the magnitude of such differences by reporting effect sizes.

This paper will determine the strength and consistency of neurobehavioral impairment in cognitive and psychomotor function in opioid exposed infants and pre-school children when compared to healthy non-opioid exposed controls by performing a systematic literature review and consequently quantitatively synthesising the existing literature using meta-analytic methodology [44,45].

## Method

### Inclusion and exclusion criteria

The systematic review of the literature was conducted accordingly to the Meta-analysis of Observational Studies in Epidemiology (MOOSE) guidelines [46] and the Preferred Reporting Items for Systematic reviews and Meta-Analysis (PRISMA) guidelines [47].

For the purpose of this review, the meanings of the terms ‘opioid’ and ‘opiate’ were considered as largely synonymous, with opioid being used, as it has a broader definition. An Infant was defined as a child up to 2 years old, pre-school child as one between 3 and 5 years of age and a school child as one between 6 and 12 years of age. Neurobehavioral function was defined as ‘growth of perceptual, emotional, intellectual, and behavioural capabilities and functioning during childhood (prior to puberty) which includes development of language, symbolic thought, logic, memory, emotional awareness, empathy, a moral sense, and a sense of identity, including sex-role identity’ [48].

Only studies that recruited opioid users were included in the meta-analysis. Furthermore all trial methodologies, not only RCTs, were considered. Studies had to use a validated diagnostic system and explicitly define whether their participants were opioid/methadone dependent [49,50].

We excluded studies that recruited mothers who were polydrug users during term pregnancy even though they might have also been taking opioids. Studies that only investigated the immediate effects of opioid use on neonates including neonatal abstinence syndrome and the neurological consequences of opioid exposure were also excluded. Sufficient study statistics not convertible to effect size (*d*) e.g. means, standard deviation, F, t, X ^18^ were also excluded, as well as studies with less than 15 in the total sample size.

### Search strategy

Articles were identified using an electronic and hand strategy based search. A computer based search was performed using the following database: Cinahl, EMBASE, PsychINFO and MEDLINE between the periods of January 1995 to January 2012 (17 years). No language constraints were applied. Subject headings originally included ‘*child, opioid, prenatal exposure and substance misuse’.* (Refer to Additional file 1: Table S1)

This was followed with the term ‘neurobehavioral’ which was subsequently replaced with a succession of terms describing names of a list of cognitive and psychomotor tests and using wild cards.

Two of the authors (AB and KA) independently reviewed all the identified abstracts from the electronic search, selected studies and published reviews. A snowballing technique was employed so that the reference list of the identified articles was screened to find other suitable studies. The literature search was further enhanced by hand searching 22 journals for the last 5 years (2008–2012). They include *Drug and Alcohol Dependence, Addictive Behaviours, Addiction, European Addiction Research, Journal of Substance Abuse Treatment, Child Neuropsychology, Neurotoxicology, Neurotoxicology and Teratology, Toxicology Letters, Psychological Medicine, European Journal of Paediatrics, Paediatrics, Developmental and Behavioral Paediatrics, Archives of Diseases in Childhood, Paediatric Research, NeuroImage, Early Human Development, Women and Birth, Obstetrics, British Journal of Gyneacology and Obstetrics, British Medical Journal, Neuroscience and Biobehavioral Reviews.*

### Data analysis and study detail

Standard meta-analytic techniques were employed to this review [51]. Magnitude is indexed with the effect size *d* that is meant to reflect the degree to which the dependent variable is present in the sample group or the degree to which the null hypothesis is false [52]. In mathematical terms *d* is the difference between two group means standardised via pooled standard deviation units. Effect sizes (i.e. Cohen’s *d* statistics) were calculated for each neurobehavioral test and then adjusted for sampling bias [53]. A value of 0.80 is regarded as a large effect size, 0.5 as a medium effect and 0.2 small [54,55]. Formulae were appropriately adjusted so that all derived statistics informally represented the same direction; that is the same polarity of performance when comparing groups. Negative scores always represented worse performance on the part of the opiate exposed group.

The multi-domain model is the most widely used model of infant-pre-school assessment. The theoretical basis of the model is that the Child Development is an interactively unfolding, continuous process that occurs in several distinct but interrelated domains. Traditionally these domains include (a) motor (fine and gross motor skills), (b) communication (receptive and expressive language), (c) cognition (problem solving skills), (d) adaptive competence (dressing, eating, toileting), and (e) personal-social competence [56,57]. For this review all relevant test variables were coded into one of three neurobehavioral domains [56,58].

1. Cognitive

2. Psychomotor

3. Behavioural observations

In keeping with recommendations on meta-analytical research in neuropsychology, previous factor-analysis, where possible, informed the placement of measures into the aforementioned domains. This approach provides an objective alternative to the arbitrary grouping of neuropsychological variables on the basis of face validity or unconfirmed notions held within the existing literature [59]. Unfortunately the factor-analytical studies to date do not encompass all of the neuropsychological measures that were encountered in this comprehensive systematic review. To this end, there was also a reliance on authoritative texts and discussion with experts in the field of neuropsychology and/or cognitive measures to help organise remaining measures [57,60] and, when necessary, we relied on the classification used by the authors of a given study [5,42,43] (Table 1).

**Table 1:** Neurobehavioral functions

**Main domain**	**Definition**	**Tests**
**General cognitive**	Child’s ability to learn and solve problems	WPPSI-R, DAS, SBIS, MSCA,MSEL, GDS, BSID
**Language**	Child’s ability to both understand and use language	PPVT-III, NEPSY, RDLS, GDS
**Non verbal processing**	Child’s ability to organize the visual-spatial field, adapt to new or novel situations, and/or accurately read nonverbal signals and cues.	K-ABC, Non verbal subtests of DAS, WPPSI, MSCA
**Psychomotor**	The child’s ability to connect thoughts with muscle movements	Vineland Motor Domain, MSCA, NEPSY, GDS, BSID
**Executive functions**	Child’s ability to analyze situations, plan and take action, focus and maintain attention, and adjust actions as needed to get the job done	WPPSI-R Animal Pegs, NEPSY, GDS
**Memory**	Child’s ability to hold and manipulate information over brief periods of time, in the course of ongoing cognitive activities	DAS, MSCA, BSID, NEPSY
**Social/emotional adjustment**	Child’s ability to interact with others, including helping themselves and self-control.	VSS, CBC, CBRS, GDS, IBR, RPD-Q, VSMS

BSID, *Bayley Scales of Infant Development Test (Motor and Cognitive);* CBRS, *Conners’ Behavior Rating Scale*; CBC, *Achenbach Child Behavior Checklist*; DAS, *Differential Ability Scale-Preschool Core*; GDS, *Griffiths Developmental Scale;* IBR, *Bayley’s Infant Behaviour Record;* K-ABC, *Kaufman Assessment Battery for Children;* MCSA, *McCarthy Scales of Children’s Abilities*; MSEL, *Mullen Scales of Early Learning;* NEPSY, Developmental NEuroPSYchological Assessment; PPVT-III, *Peabody Picture Vocabulary Test-3*^*rd*^*Edition*; RPD-Q, *Rapid Pre-screening Denver Questionnaire;* RDLS, *Reynell Developmental Language Scale*; SBIS, *Stanford-Binet Intelligence Scale;* VSS*, Vineland Social-Emotional Early Childhood Scales*; VSMS, *Vineland Social Maturity Scale*; WPPSI-R, *Weschler Preschool and Primary Scale of intelligence-Revised.*

To meet the assumption of independence, when multiple test variables in a study contributed to any one neuropsychological domain, the effect size for each measure was assessed separately and then the mean effect size of these measures were combined to assess the overall outcome in the respective area of functioning. Multiple measurements can increase the likelihood of Type 1 errors and so a *p* value over 0.01 will be interpreted with caution even though analysis will use a significant level of *p < 0.05*.

Tests for the presence and degree of heterogeneity were conducted using the Q statistic [55] and I^2^ index [61] respectively. However, quantification of heterogeneity is only one component of a wider investigation of variability across studies, the most important being diversity in clinical and methodological domains, and the observed degree of inconsistency across studies with regards to the direction of effects [62]. As different scales were sometimes used by different studies, standardised mean difference (SMD) effect-size estimates were calculated. In case of significant heterogeneity, random effect models were applied [63,64].

Research with statistically significant results are potentially more likely to be submitted and published than studies with non-significant results. The presence of such publication bias was assessed informally by visual inspection of funnel plots and formally by its statistical analogue, Fail Safe N, according to Orwin [65].

A Fail-Safe N is the number of non significant, unpublished, or missing studies that would need to be added to the meta-analysis in order to change the overall result from significance to non-significance. More than two studies are needed to enable a Fail-Safe N to be calculated.

Eligible research studies comprising a common dependent variable as well as statistics that can be transformed into effect sizes were systematically surveyed. Individual study results (typically means and standard deviations from each group) and relevant moderator variables considered as relevant by previous reviews (e.g. dosage of maternal methadone during pregnancy, gestational age, presence of Neonatal Abstinence Syndrome (NAS), quality of the study, and population studied) were used as moderators if needed during this review. They were abstracted, quantified,coded and assembled into a database and analysed using Comprehensive Meta-Analysis Version 2 [66]*.* The significance level was *p* = 0.01 and in Q statistics *p* = 0.10.

### Assessment of study quality

For all review questions, data were extracted by one reviewer and checked by another. Discrepancies were resolved by referral to the original studies and, if necessary, arbitration done by a third reviewer. Duplicate publications were actively screened for and, when found, the latest and most complete report was used. The Effective Public Health Practice Project (EPHPP) quality assessment checklist (amended) was used in this study [67]. For pragmatic reasons no papers were excluded on quality grounds as all papers were weak to moderate (Refer to Additional file 2: Table S2).

Ethical approval and informed patient consent was not required as this study was a literature review and had no direct patient contact or influence on patient care.

## Results

### Studies selected and population studied

Combined searches yielded 1452 references. In total 65 articles were retrieved for further assessment from which 65 studies identified intrauterine exposure to opioids and reported health and developmental outcomes for the opioid exposed children. From these studies, 8 articles were found to investigate the cognitive, psychomotor and behavioral outcomes in opioid exposed infants, pre-school and school children when compared to healthy non-opioid exposed controls. Only 5 studies could be further utilised in this meta-analysis since 1 study measured motor rather than psychomotor skills [68] and 2 other studies had small sample sizes of < 15 [69,70].

**Figure 1:**
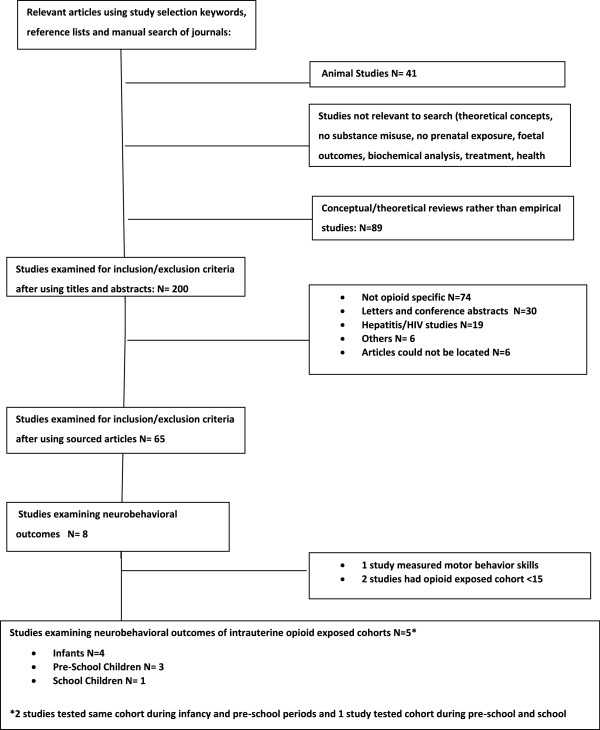
Neurobehavioral consequences of chronic opioid intrauterine exposure in infants, preschool and school children: QUality Of Reporting Of Meta-analysis (QUOROM).

Furthermore 2 studies tested the same cohort during infancy and pre-school periods [6,71] and another study tested the cohort during pre-school and school periods [72]. Considering that only one study measured outcomes during the school period [72] it was decided that further analysis should concentrate on the infancy and pre-school periods. During these periods there were 4 studies comparing opioid exposed infants with controls [6,71,73,74] and 3 studies comparing opioid exposed pre-school children with controls [6,71,72]. In Hans et al. [74] infants in the cohort were tested at 1 and 2 years old allowing two observational points (Figure 1).

All studies were case controlled observational studies conducted with a population living in urbanised and low socioeconomic communities exposed to either heroin or methadone.

The global quality assessment reported four studies as of moderate quality and one as weak. The assessment for analysis performed was moderate for all studies but all other domains were reported as either weak or moderate (see Additional file 2: Table S2).

Cohort characteristics for the 4 studies comparing opioid exposed infants with controls describe a total number of 218 individuals tested compared to a total of 205 non opioid exposed controls. The mean infant age was 14.1 months (1.2 years). Cohort characteristics for the 3 studies comparing opioid exposed pre-school children with non opioid exposed controls describe a total number of 224 individuals tested compared to a total of 231 non opioid exposed controls. The mean age of the pre-school children tested was 50.7 months (4.2 years). General and specific characteristics of the included studies are shown on Tables 2&3.

**Table 2:** General characteristics of selected studies comparing opioid exposed infants and children with non opioid exposed controls (n = 5)

**Study**	**Age in months when tested**	**Number**	**Country**	**Type of opioid exposure**	**Socio-economic status**	**Measures used (Neurobehavioral)**
**Infants**						
Hunt *et al.*[6]	19.9	79OE	Australia	Illicit heroin	n/a	VSMS, MSCA, BSID
		61C				
Moe *et al.*[71]	12	64OE	Norway	Illicit heroin	3.9OE*^1^ v 4.1C	MSCA, BSID
		52C				
Hans *et al.*[74]	12/24	33OE	USA	Illicit heroin	5*^2^	BSID, IBR
		45C		Prescribed methadone		
Bunikowski *et al.*[73]	12.4	42OE	Germany	Illicit heroin	4OE*^3^ v 6C	GDS, RPD-Q
		47C		Prescribed methadone		
**Pre-school children**						
Hunt *et al.*[6]	38.2	67OE	Australia	Illicit heroin	n/a	BSID, MSCA
		44C				
Moe *et a*l. [71]	54	64OE	Norway	Illicit heroin	3.9OE*^1^ v 4.1C	BSID, MSCA
		52C				
Ornoy *et al.*[72]	60	93OE	Israel	Illicit heroin	3.9OE*^3^	BSID, MCSA, Achenbach CBC
		50EC			2.4C	
		85C				

OE, *Opioid Exposed*; EC, *Environmental Controls*; C, *Controls*; *1, *SES or mean value of both parent education and occupation*; *2, *Hollinghead level*; *3, *Socio-status*; n/a, not available. Achenbach CBC, *Achenbach Child Behavior Checklist (Externalising including attention)*; BSID, *Bayley Scales of Infant Development Test (Psychomotor and Mental Scores)*; GDS, *Griffiths Developmental Scale (Locomotor)*; IBR, *Bayley’s Infant Behaviour Record;* MCSA, *McCarthy Scales of Children’s Abilities (Motor scale)*; RPD-Q, *Rapid Pre-screening Denver Questionnaire;* VSMS, *Vineland Social Maturity Scale.*

**Table 3:** Specific characteristics of selected studies comparing opioid exposed infants and children with non opioid exposed controls (n = 5)

**Study**	**Age in months when tested**	**Gestational age in weeks**	**Mean birth weight in g**	**Mean head circumference (cms)**	**Mother’s age (median) in years**	**Mother’s methadone dose in mg**
**Infants**						
Hunt *et al.*[6]	19.9	37.7	2900in OE	33.6 in OE	n/a	n/a
			3300 in C	34.5 in C		
Moe *et al.*[71]	12	n/a	3037 in OE	34 in OE	n/a	na
			3754 in C	35.8 in C		
Hans *et al.* (2011)	12/24	n/a	2922 in OE	n/a	27.1 in OE	20 mg
			3236 in C		25.8 in C	
Bunkowski *et al.* (1998)	12.4	37.4	2783 in OE	n/a	27 in OE	n/a
			3240 in C		30.2 in C	
**Pre-school children**						
Hunt *et al.*[6]	38.2	37.7	2900in OE	33.6 in OE	n/a	n/a
			3300 in C	34.5 in C		
Moe *et al.*[71]	54	n/a	3037 in OE	34 in OE	n/a	n/a
			3754 in C	35.8 in C		
Ornoy *et al.*[72]	60	36	2487 in OE	27.8 in OE	n/a	n/a
			3346 in C	39.8 in C		

Mg, *Milligrammes*; g, *Grammes*; cms, *Centimetres*; n/a, *Not available;* OE, *Opioid Exposed;* C*, Non-Opioid Exposed Controls.*

### Neurobehavioral function

There were six effect size measures possible (3 for the infant cohort and 3 for the pre-school cohort groups) from the selected studies. There were no effect sizes identified as greater than 2× inter-quartile range (25 and 75 percentile) from the nearest quartile (outliers) [75].

#### Opioid exposed infants compared with non-opioid exposed infants

**Figure 2:**
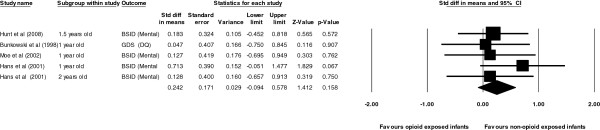
Forrest plots comparing cognition in opiod and non-opiod exposure.

For cognition: a total of four studies were pooled including 251 opioid exposed and 315 non-opioid exposed infants. Pooling of the four studies revealed a non significant effect size of 0.24 in favour of non-opioid exposed controls. (Z = 1.41, *p =* 0.16). The Q and I^2^ statistics, showed no significant evidence of heterogeneity with the use of a fixed effects model (Q = 1.88, *p <* 0.76, I^2^ = 0.00). Lastly the 95% confidence interval did contain zero and, hence the null hypothesis that the effect size was not different from zero could not be rejected (95% CI; -0.09, 0.58) (Figure 2).

**Figure 3:**
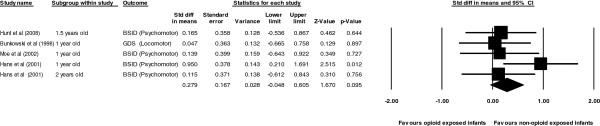
Forrest plots comparing psychomotor in opiod and non-opiod exposure.

For psychomotor: a total of four studies were pooled including 251 opioid exposed and 315 non-opioid exposed infants. Pooling of the four studies revealed a non significant effect size of 0.28 in favour of non-opioid exposed controls. (Z = 1.67, *p =* 0.09).The Q and I^2^ statistics, showed no significant evidence of heterogeneity with the use of a fixed effects model (Q = 3.98, *p <* 0.41, I^2^ = 0.00). Lastly the 95% confidence interval did contain zero and, hence the null hypothesis that the effect size was not different from zero could not be rejected (95% CI; -0.05, 0.61) (Figure 3).

**Figure 4:**
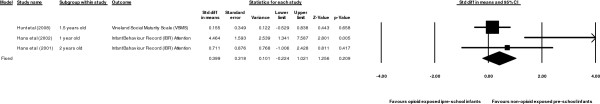
Forrest plots comparing behaviour in opiod and non-opiod exposure.

For behaviour: a total of three studies were pooled including 145 opioid exposed and 216 non-opioid exposed infants. Pooling of the three studies revealed a non significant effect size of 0.40 in favour of non-opioid exposed controls. (Z = 1.25, *p =* 0.20). The Q and I^2^ statistics, however showed significant evidence of heterogeneity with the use of a fixed effects model (Q = 7.13, *p <* 0.03, I^2^ = 71.93). As a result an additional analysis was performed that corrected for random effects. The corrected mean effect size changed to 1.21 and a non-significant Z score (Z = 1.30, *p =* 0.19). Lastly the 95% confidence interval did contain zero and, hence the null hypothesis that the effect size was not different from zero could not be rejected (95% CI; -0.61, 3.03) (Figure 4).

#### Opioid exposed pre-school children compared with non-opioid exposed pre-school children

**Figure 5:**
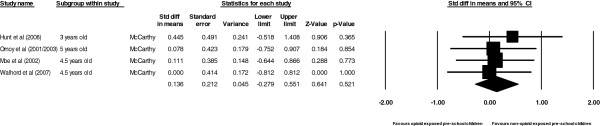
Forrest plots comparing cognition in opiod and non-opiod exposure.

For cognition: a total of three studies were pooled including 224 opioid exposed and 181 non-opioid exposed pre-school children. Pooling of the three studies revealed a non significant effect size of 0.18 in favour of non-opioid exposed controls. (Z = 0.75, *p =* 0.46).The Q and I^2^ statistics, showed no significant evidence of heterogeneity with the use of a fixed effects model (Q = 0.38, *p <* 0.83, I^2^ = 0.00). Lastly the 95% confidence interval did contain zero and, hence the null hypothesis that the effect size was not different from zero could not be rejected (95% CI; -0.30, 0.67) (Figure 5).

**Figure 6:**
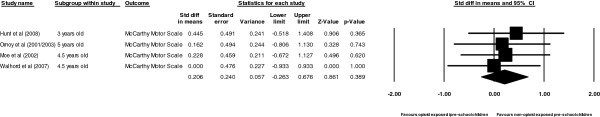
Forrest plots comparing psychomotor in opiod and non-opiod exposure.

For psychomotor: a total of three studies were pooled including 224 opioid exposed and 181 non-opioid exposed pre-school children. Pooling of the three studies revealed a non significant effect size of 0.28 in favour of non-opioid exposed controls. (Z = 1.00, *p =* 0.32).The Q and I^2^ statistics, showed no significant evidence of heterogeneity with the use of a fixed effects model (Q = 0.18, *p <* 0.91, I^2^ = 0.00). Lastly the 95% confidence interval did contain zero and, hence the null hypothesis that the effect size was not different from zero could not be rejected (95% CI;-0.27, 0.82) (Figure 6).

**Figure 7:**
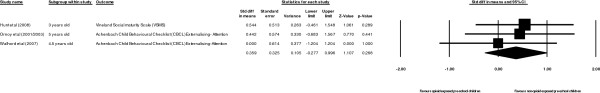
Forrest plots comparing behaviour in opiod and non-opiod exposure.

For behaviour: a total of two studies were pooled including 160 opioid exposed and 129 non-opioid exposed pre-school children. Pooling of the two studies revealed a non significant effect size of 0.38 in favour of non-opioid exposed controls. (Z = 1.30, *p =* 0.19). The Q and I^2^ statistics, showed no significant evidence of heterogeneity with the use of a fixed effects model (Q = 0.02, *p <* 0.89, I^2^ = 0.00). Lastly the 95% confidence interval did contain zero and, hence the null hypothesis that the effect size was not different from zero could not be rejected (95% CI;-0.25, 1.25) (Figure 7).

## Discussion

### Key findings

In this first ever quantitative review of the research literature on the neurobehavioral outcomes as a result of intra-uterine opioid exposure in infants and pre-school children the meta-analysis has determined what abilities, if any, were reliably found impaired across studies when compared with non-opioid exposed controls. Our findings indicate no significant impairments in cognitive, psychomotor or observed behavioural outcomes for chronic intra-uterine exposed infants and pre-school children, although in all domains there is a trend to poor outcomes in infants/children of opioid using mothers (Table 4).

**Table 4:** Effect sizes and associated statistics for neurobehavioral domains in opioid exposed infants and pre-school children compared to others who have no history of opioid (or any other illicit and/or alcohol use) exposure during pregnancy

Neuropsychological Domains*	**Studies**^**1**^	**Effect Size**^**2**^	**SE **^**3**^	**N **^**4**^	**Lower Limit **^**5**^	**Upper Limit **^**6**^	**Q **^**7**^	***p f*** **or Q **^**8**^	**Z **^**9**^	***p *** **for Z **^**10**^	**I**^**2**^^**11**^	**Fail safe N **^**12**^
**Infants**												
**Cognition**	4	0.24	0.17	251	-0.09	0.58	1.88	*0.76*	1.41	*0.16*	0.00	0
**Psychomotor**	4	0.28	0.17	251	-0.05	0.61	3.98	*0.41*	1.67	*0.09*	0.00	5
Behaviour*^1^	3	1.21	0.93	145	-0.61	3.03	7.13	*0.03*	1.30	*0.19*	71.93	2
**Pre-school children**												
**Cognition**	3	0.18	0.25	224	-0.30	0.67	0.38	*0.83*	0.75	*0.46*	0.00	0
**Psychomotor**	3	0.28	0.28	224	-0.27	0.82	0.18	*0.91*	1.00	*0.32*	0.00	0
**Behaviour**	2	0.50	0.38	160	-0.25	1.25	0.02	*0.89*	1.30	*0.19*	0.00	np

^1^ = *Number of studies used to calculate effect size*, ^2^ = *Cohen’s d effect size*, ^3^ = *Standard Error*, ^4^ = *Total number of subjects in opioid exposed cohort* ,^5^ = *Lower limit of the 95% confidence interval for the effect size,*^6^ = *Upper limit of the 95% confidence interval for the effect size*, ^7^ = *Q statistic: A test of homogeneity*, ^8^ = *Probability that Q statistics significantly diff than 0*, ^9^ = *One sample Z Statistic*, ^10^ = *Probability that Z Statistics, is significantly diff than 0*, ^11^ = *I*^*2*^*statistics,*^12^ = *Fail Safe N: a measure of publication bias,* n/p = not possible since one needs more than 2 studies to perform this analysis, ^*^ All neuropsychological domains had random effects models employed except*^1^ where random effect model employed.

The result of this systematic review is in accordance with Whitham [42] who conducted an open label non randomised flexible dosing longitudinal study with results showing that children prenatally exposed to illicit heroin and/or methadone did not differ to non-exposed infants and other children in cognitive, psychomotor and caregiver rated temperament outcomes. As a recent review observed, the conflicting results of traditional systematic reviews on this subject could be that most children in these studies were exposed to other drugs in addition to opioids such as methadone [5]. Another explanation given to the conflicting results may be that various studies were conducted using different neurobehavioral tests at different ages of development. Prenatal opioid exposure may affect children’s cognitive and psychomotor performance differently at different ages resulting in neurobehavioral outcomes that might improve or worsen over time [5]. This analysis could only be conducted during the two early stages of a child’s development (infancy and pre-school children). It was not possible to conduct a similar analysis on children aged between 6–12 years due to the presence of only one study meeting the criteria for analysis.

The studies included in the meta-analysis had differences in the measurement of exposure to opiates. Most studies involved the use of illicit heroin wherein the dosage is highly variable and based on a large number of factors, while two of the studies involved methadone prescribed in a controlled environment. None of the studies commented on the use of illicit drugs and alcohol which may also have a bearing on the outcomes of interest.

### Limitations

The results of our analysis must be cautiously interpreted bearing its limitations in mind. The inclusion criteria used for this meta-analysis was very stringent so as to exclude neurobehavioral effects as a result of intra-uterine maternal polydrug use and the potential confusion of neurobehavioral outcomes associated with the neonatal abstinent syndrome and other opioid withdrawal presentations during the neonatal period (Jones et al., 2010). The main limitation of using a meta-analytic technique is the small number of primary studies available for analysis and also their small sample size. This limits the generalisability of the result [75] and the small sample size of individual studies means they may miss an increased risk of the occurrence of relatively rare outcomes like ADHD, autism or psychosis. The quality assessment of the individual studies would also raise some concerns about the generalisability of the findings as all were reported as of moderate or weak quality.

Meta-analysis tends to present results as composite scores for broad neurobehavioral functions using different neuropsychological tests. This is a convenient way to summarise findings but it combines data from tests potentially exploring different neuropsychological processes (e.g. memory tests assessing immediate or delayed recall, learning or recognition) and possibly generating results of questionable theoretical relevance. This study attempted to minimise this by utilising neuropsychological domains agreed by consensus and used in systematic reviews on chronic substance use effects [24,76].

Even though the meta-analysis grouped together studies that used the same rating scales on a cohort who clearly were exposed to opioids, it was not possible from the information presented in the studies to exclude other confounding effects such as dosage of maternal opioid use during pregnancy [5], timing during pregnancy when the fetus was exposed to opioids with potential resulting behavioral teratogenicity [77], the differential effects of exposure to different opioids [42] and/or gender specific neurobehavioral influences [71], other illicit drugs including cocaine and alcohol use during pregnancy [5]. Each included study reported that there were no polydrug using mothers within their cohorts but it is not possible to be certain that this was achieved.

### Clinical relevance

This meta-analysis helps in supporting certain clinical observations in this population. The observed, if any, neurobehavioral outcomes in infants and pre-school children prenatally exposed to opioids are very often attributed to substance exposure. However it is important to examine the contribution of other influences on a child’s development. Ongoing maternal depressive illness is correlated with poorer cognitive and motor development and increase in teacher and parent rated behavior problems in pre-school children [78,79]. Poverty and low socio-economic status is inversely related to children’s developmental performance [80,81]. A study examining the relationship between birth weight and cognitive functioning among children in South Australia indicated that cognition at 2 years of age was significantly related to birth weight [82]. However the magnitude of the association attenuated over time became non-significant in childhood. Factors that became significantly associated with neurobehavioral outcomes included low socio-economic status, low maternal IQ, poor quality of the home environment and child’s lead exposure. Overall it is increasingly becoming evident that the risk factors that can predict poor neurobehavioral outcomes is not the drug fuelled lifestyle or actual substance exposure during pregnancy but the presence of multiple, inter-related and weighted variables cumulatively influencing neurobehavioral outcomes [3]. The risk factors were: maternal mental health, maternal attitudes toward parenting and maternal child–parent interaction, maternal education, parental occupation, minority status, stressful life events and family size with not one risk factor contributing exclusively to one cognitive or other neurobehavioral outcomes.

In many countries, including the UK, pharmacological maintenance with methadone is the first line of treatment for pregnant opioid dependent women [17,83]. Whilst treatment with methadone during pregnancy results in fewer complications for both mother and infant when compared with the use of illicit opioids such as heroin, its use in pregnancy is associated with high rates of neonatal abstinence syndrome [84,85]with its treatment involving using another opioid, morphine [86]. Prenatal exposure to opioids also significantly increases the risk of low birth weight and small head circumference as shown in the cohort of children in the studies selected for this review. However this analysis did not observe any increased risk in neurobehavioral problems in opioid exposed infants and pre-school children compared to non-exposed peers suggesting that there is no neurobehavioral sequelae to the chronic prenatal and, if treated for NAS with opioids, also postnatal, exposure to opioids with any effects being short term and/or reversible.

## Conclusion

Chronic intra-uterine opioid exposed infants and pre-school children experience no significant impairment in neurobehavioral outcomes when compared to non-exposed peers, although in all domains there was a trend to poorer outcomes. Interpretation of this meta-analysis needs to appreciate the heterogeneous population studied, the limited number of studies analysed due to the stringent inclusion criteria and the small numbers within the individual studies. Additional studies are needed to improve the power of a future meta-analysis to produce significant results. And longitudinal studies are needed to determine if any neuropsychological impairments appear after the age of 5 years and to help investigate further the role of environmental risk factors on the effect of ‘core’ phenotypes.

## Competing interests

The authors declare that they have no competing interests.

## Authors’ contributions

AB, CMC & DJP conceived the study and participated in its design and coordination KA carried out the systematic review supported by the other authors. AB performed the statistical meta-analysis and wrote the first draft of the manuscript. All authors read and approved the final manuscript.

## Authors’ information

Dr Alex Baldacchino^*^: MD, FRCPsych, MPhil, PhD. Clinical Senior Lecturer (University of Dundee) and Consultant Psychiatrist (NHS Fife), Division of Neuroscience, Medical Research Institute, University of Dundee, Ninewells Hospital and Medical School, Dundee, DD1 9SY, UK

Ms Kathleen Arbuckle: BSc, MPH. ESRC/MRC PhD student (University of Dundee), Division of Health Population, Medical Research Institute, University of Dundee, Ninewells Hospital and Medical School, Dundee, DD1 9SY, UK

Dr Dennis J Petrie: BEcon, BSc, PhD. Senior Research Fellow, Centre for Health Policy, , Melbourne School of Population and Global Health, University of Melbourne, Victoria, Australia

Dr Colin McCowan: BSc, MSc, PhD. Reader in Health Informatics, Robertson Centre for Biostatistics, Institute of Health and Wellbeing, College of Medical, Veterinary and Life Sciences, University of Glasgow, Boyd Orr Building, Level 11, Glasgow, G12 8QQ, UK

## Pre-publication history

The pre-publication history for this paper can be accessed here:

http://www.biomedcentral.com/1471-244X/14/104/prepub

## Supplementary Material

Additional file 1: Table S1 Mesh terms used and searches conducted. Click here for file

Additional file 2: Table S2 Study quality. Click here for file
