# Erratum: Neurobehavioral consequences of chronic intrauterine opioid exposure in infants and preschool children: a systematic review and meta-analysis

**DOI:** 10.1186/s12888-015-0438-5

**Published:** 2015-06-25

**Authors:** Alex Baldacchino, Kathleen Arbuckle, Dennis J Petrie, Colin McCowan

**Affiliations:** Division of Neuroscience, Medical Research Institute, University of Dundee, Ninewells Hospital and Medical School, Dundee, DD1 9SY UK; Division of Population Health Science, Medical Research Institute, University of Dundee, Ninewells Hospital and Medical School, Dundee, DD1 9SY UK; Centre for Health Policy, Melbourne School of Population and Global Health, University of Melbourne, Victoria, Australia; Robertson Centre for Biostatistics, Institute of Health and Wellbeing, College of Medical, Veterinary and Life Sciences, University of Glasgow, Boyd Orr Building, Level 11, Glasgow, G12 8QQ UK

## Correction

After publication of this work [1] we became aware that during our entry of raw data into the Complementary Meta-Analysis (CMA) programme we transposed one of the columns of data. This meant that the values generated by all of the meta-analysis and results produced in the published manuscript including those displayed Figures two to seven (Figures 1, 2, 3, 4, 5, 6 here) and Table four (Table 1 here) were incorrect. We subsequently repeated the meta-analysis and updated the Figures, Table and manuscript to reflect the new results following this re-analysis.

**Figure 1:**
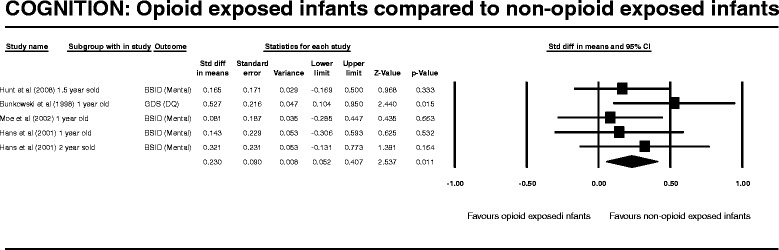
**COGNITION: Opioid exposed infants compared to non-opioid exposed infants.**

**Figure 2:**
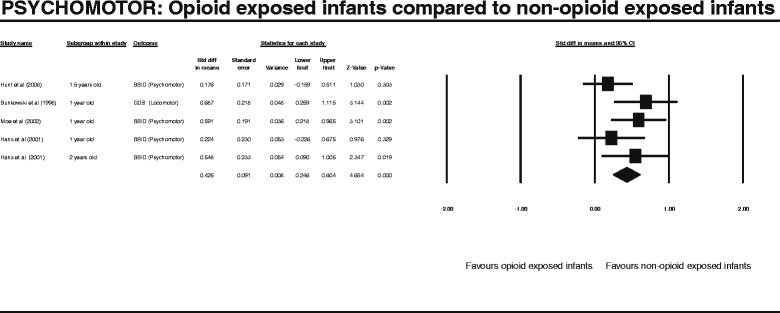
**PSYCHOMOTOR: Opioid exposed infants compared to non-opioid exposed infants.**

**Figure 3:**
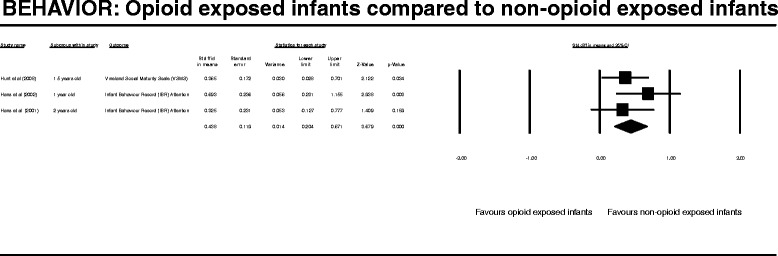
**BEHAVIOR: Opioid exposed infants compared to non-opioid exposed infants.**

**Figure 4:**
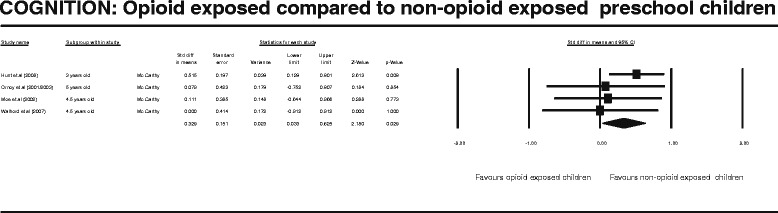
**COGNITION: Opioid exposed compared to non-opioid exposed preschool children.**

**Figure 5:**
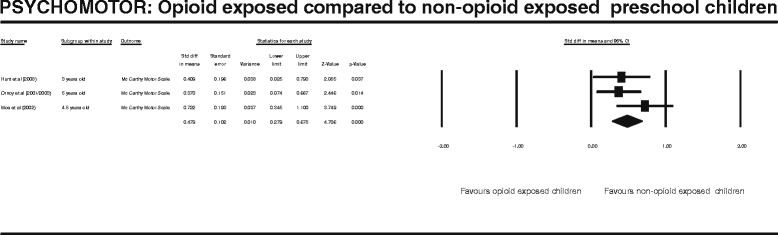
**PSYCHOMOTOR: Opioid exposed compared to non-opioid exposed preschool children.**

**Figure 6:**
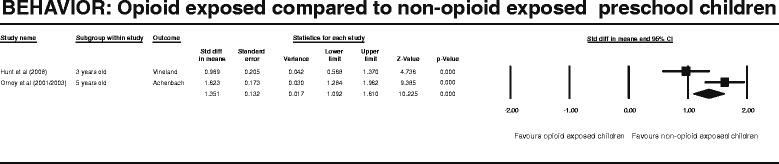
**BEHAVIOR: Opioid exposed compared to non-opioid exposed preschool children.**

**Table 1:** **Effect sizes and associated statistics for neurobehavioral domains in intrauterine opioid exposed infants and preschool children compared to others who have no history of intrauterine opioid exposure during pregnancy**

Neuropsychological domains*	**Studies¹**	**Effect size²**	**SE ³**	**N** ^**4**^	**Lower limit** ^**5**^	**Upper limit** ^**6**^	**Q** ^**7**^	***p f*** **or Q** ^**8**^	**Z** ^**9**^	***p*** **for Z** ^**10**^	**I²** ^**11**^	**Fail safe N** ^**12**^
**INFANTS**												
**Cognition**	4	0.23	0.09	251	0.05	0.41	2.97	*0.56*	2.54	*0.01*	0.00	4
**Psychomotor**	4	0.43	0.09	251	0.25	0.60	5.36	*0.25*	4.65	*0.00*	25.41	25
**Behavior**	3	0.44	0.12	145	0.20	0.67	1.59	*0.45*	3.68	*0.00*	0.00	8
**PRESCHOOL CHILDREN**												
**Cognition**	3	0.33	0.15	224	0.03	0.63	2.19	*0.53*	2.18	*0.03*	0.00	0
**Psychomotor**	3	0.58	0.15	224	0.28	0.88	6.36	*0.09*	3.78	*0.00*	52.84	31
Behavior*1	2	1.31	0.33	160	0.67	1.95	5.96	*0.02*	3.99	*0.00*	83.21	np

^1^= *Number of studies used to calculate effect size*, ^2^= *Cohen’s d effect size*, ^3^= *Standard Error*, ^4^=*Total number of subjects in opioid exposed cohort* ,^5^= *Lower limit of the 95% confidence interval for the effect size,* ^6^= *Upper limit of the 95% confidence interval for the effect size*, ^7^= *Q statistic: A test of homogeneity*, ^8^= *Probability that Q statistics significantly diff than 0*, ^9^= *One sample Z Statistic*, ^10^= *Probability that Z Statistics, is significantly diff than 0*, ^11^= *I² statistics,* ^12^ *= Fail Safe N: a measure of publication bias,* n/p= not possible since one needs more than 2 studies to perform this analysis, * All neuropsychological domains had fixed effects model employed except *1 where a random effect model was employed.

The new conclusions of the paper show significant impairments, at a significance level of *p < 0.05*, for cognitive, psychomotor and observed behavioral outcomes for chronic intrauterine opioid exposed infants and/or preschool children compared to non-opioid exposed infants and children. This is in contrast to a non significant trend to poorer outcomes for chronic intrauterine opioid exposed infants and/or preschool children that we originally reported.

We regret any inconvenience that this inaccuracy in the data presented in the original manuscript might have caused. We wish to thank Dr Egil Nygaard for bringing this matter to our attention.
