# Supplementary material for: Neurobehavioral consequences of chronic intrauterine opioid exposure in infants and preschool children: a systematic review and meta-analysis
Source: BMC Psychiatry. 2014 Apr 8;14:104. doi: 10.1186/1471-244X-14-104 (PMC4021271; doi:10.1186/1471-244X-14-104)
Supplement: Additional file 1: Table S1 — Mesh terms used and searches conducted. [file 1471-244X-14-104-S1.doc]

# Supplementary Tables

# Additional file 1: Table S1: Mesh terms used and searches conducted

**1. Child**

1. exp Child/

2. limit 1 to (yr="1995 -Current" and ("all infant (birth to 23 months)" or "preschool child (2 to 5 years)" or "child (6 to 12 years)"))

**2. Opioid**

1. exp Analgesics, Opioid/

2. limit 1 to yr="1995 -Current"

**3. Prenatal exposure**

1. exp Child/ or exp Pregnancy/ or exp Fetus/ or exp Prenatal Exposure Delayed Effects/ or exp Maternal-Fetal Exchange/

2. limit 1 to yr="1995 -Current"

**4. Substance misuse**

1. exp Alcoholism/ or exp Substance-Related Disorders/ or substance misuse.mp. or exp Social Work/

2. limit 1 to yr="1995 -Current"

**5**. 1 or 3

**6.** 2 or 4

**7.** 6 and 5

**8.** 4 and 7
