# Supplementary material for: Neurobehavioral consequences of chronic intrauterine opioid exposure in infants and preschool children: a systematic review and meta-analysis
Source: BMC Psychiatry. 2014 Apr 8;14:104. doi: 10.1186/1471-244X-14-104 (PMC4021271; doi:10.1186/1471-244X-14-104)
Supplement: Additional file 2: Table S2 — Study quality. [file 1471-244X-14-104-S2.doc]

Additional file 2: Table S2: Study Quality

| **Study** | **Selection Bias** | **Design** | **Confounders** | **Data Collection Methods** | **Withdrawals and Drop outs** | **Analyses** | **Global Rating** |
| --- | --- | --- | --- | --- | --- | --- | --- |
| Bunkowski *et al.* (1998) | W | M | M | W | W | M | M |
| Hans *et al.* (2011) | M | W | M | M | M | M | M |
| Hunt *et al.* (2008) | M | W | M | M | W | M | M |
| Moe *et al.* (2002) | M | W | W | M | W | M | W |
| Ornoy *et al.* (2001) | W | M | M | M | M | M | M |

M= Moderate, W=Weak
